# Can't do it, won't do it! Developing a theoretically framed intervention to encourage better decontamination practice in Scottish dental practices

**DOI:** 10.1186/1748-5908-4-31

**Published:** 2009-06-05

**Authors:** Debbie Bonetti, Linda Young, Irene Black, Heather Cassie, Craig R Ramsay, Jan Clarkson

**Affiliations:** 1 Dental Health Services Research Unit, University of Dundee, MacKenzie Building, Kirsty Semple Way, Dundee, DD2 4BF, UK; 2 National Health Service Education for Scotland (NES), Dundee Dental Education Centre, Small's Wynd, Dundee, DD1 4HN, UK; 3 Health Services Research Unit, Health Services Building, University of Aberdeen, Foresterhill, Aberdeen, AB25 2ZD, UK

## Abstract

**Background:**

Guidance on the cleaning of dental instruments in primary care has recently been published. The aims of this study are to determine if the publication of the guidance document was enough to influence decontamination best practice and to design an implementation intervention strategy, should it be required.

**Methods:**

A postal questionnaire assessing current decontamination practice and beliefs was sent to a random sample of 200 general dental practitioners.

**Results:**

Fifty-seven percent (N = 113) of general dental practitioners responded. The survey showed large variation in what dentists self-reported doing, perceived as necessary or practical to do, were willing to do, felt able to do, as well as what they planned to change. Only 15% self-reported compliance with the five key guideline-recommended individual-level decontamination behaviours; only 2% reported compliance with all 11 key practice-level behaviours. The results also showed that our participants were almost equally split between dentists who were completely unmotivated to implement best decontamination practice or else highly motivated. The results suggested there was scope for further enhancing the implementation of decontamination guidance, and that an intervention with the greatest likelihood of success would require a tailored format, specifically targeting components of the theory of planned behaviour (attitude, perceived behavioural control, intention) and implementation intention theory (action planning).

**Conclusion:**

Considerable resources are devoted to encouraging clinicians to implement evidence-based practice using interventions with erratic success records, or no known applicability to a specific clinical behaviour, selected mainly by means of researchers' intuition or optimism. The methodology used to develop this implementation intervention is not limited to decontamination or to a single segment of primary care. It is also in accordance with the preliminary stages of the framework for evaluating complex interventions suggested by the medical research council. The next phases of this work are to test the intervention feasibility and evaluate its effectiveness in a randomised control trial.

## Background

It is estimated that in excess of 180 million instruments are re-processed in Scottish general dental practices per annum [1]. Decontamination is the combination of processes (including washing, disinfection, and sterilization) employed to make re-usable items safe for handling by users and for use on patients. Inadequately decontaminated instruments increase the risk of transmission of bacterial, viral, and fungal infections to both users and patients, including Methicillin Resistant *Staphylococcus aureus*, HIV, hepatitis B, hepatitis C, and variant Creutzfeldt-Jakob Disease [1-4]. In May 2007, the Scottish Dental Clinical Effectiveness Programme (SDCEP) published guidance on the cleaning of dental instruments specifically for dental teams working in primary care [5].

However, it is well documented that the translation of guideline recommendations into clinical practice can be a haphazard process [6-8]. The first aim of this study was to determine if the publication of the guidance document was enough to encourage the implementation of best decontamination practice. Although the funding limits of this study precluded examining what dentists were actually doing, it was posited that a gap between self-reported current and best decontamination practice, accompanied by a lack of plan to change current practice, would suggest that further intervention to encourage the implementation of best decontamination practice was needed.

The second aim of this study was to design an implementation intervention strategy, should it be required. Strategies employed to encourage the implementation of other guidelines have been aimed at individuals (e.g. audit and feedback, reminders, outreach visiting), organisation of care (e.g. case management, revision of roles, continuous quality improvement), and financial and regulatory incentives. However, these implementation interventions and their development tend to be sketchily described, and similar strategies have resulted in a range of effect sizes [9-11]. This makes it extremely difficult to choose or replicate interventions.

Literature reviews suggest that the main problem in this area may be a lack of understanding or description of the mechanism by which these interventions are achieving their effect [12-15]. Because implementing guidelines often require clinicians to change their behaviour, it may be helpful to base interventions on explanatory frameworks explicitly concerned with behaviour change. Psychological frameworks explain behaviour in terms of predictive beliefs that can be influenced, as well as methods for measuring and influencing them. In effect, they provide a means of focusing the design of an intervention and include an explanation of how it will work.

One such model is the theory of planned behaviour (TPB) [16,17]. In the TPB, the main components proposed to influence behaviour are: motivation to perform a behaviour (behavioural intention), perceived behavioural control (PBC, assessed in terms of perceived difficulty of performing the behaviour), attitude toward the behaviour, and perceptions of social pressure to perform the behaviour (subjective norm). The TPB predicts an individual is more likely to follow best decontamination practice if they intend to do so, and that they are more likely to intend to do so if they believe that they are able to overcome likely barriers (high PBC), if they think that doing so will result in consequences that they value (positive attitude), and if they believe that other people they respect want them to (positive subjective norm). These variables are all modifiable and so provide the possible targets of an intervention based on this model. Nevertheless, while this model has successfully predicted other evidence-based dental behaviours [18,19], it is not known if its components are sensitive to decontamination practice, and so if it is an appropriate one to use as the basis of an intervention to influence the implementation of the decontamination guidance. This study explored this issue in order to inform the implementation intervention strategy development.

## Methods

This was a cross-sectional study. Participants were general dental practitioners (GDPs) across Scotland. Data collection was by postal survey. The Scottish Multicentre Research Ethics Committee considered the study as a dental service audit and ethical approval was not required.

### Measures

#### Primary outcome measure: decontamination practice

A list of behaviours (Table 1), derived from the SDCEP guidance document as essential to best decontamination practice, was developed in consultation with members of the committee involved in developing the SDCEP guidance material, National Health Service Education for Scotland (NES) personnel involved in delivering post-graduate decontamination education courses and academic dentists from the University of Dundee involved in primary care dental research. Because the list included behaviours that could only be performed by the dentist, as well as behaviours that could be performed by anyone in the dental practice, two subscales as well as a total measure were assessed.

**Table 1:** Outcome measure showing best decontamination practice behaviours derived from SDCEP Guidance document

Dentist-level behaviours	1. Remove hand and wrist jewellery at the start of each session
	2. Clean hands before putting on gloves
	3. Change gloves before seeing **each **patient
	4. Use single use items only once
	5. Work in a clutter – free environment
Practice-Level Behaviours (anyone in the practice may perform)	6. Decontamination equipment (*e.g*., Washer-disinfectors, ultrasonic cleaners, sterilizers) is used in accordance with the manufacturers' instructions
	7. Testing of decontamination equipment takes place at the correct intervals
	8. Decontamination activities take place in a dirty to clean workflow
	9. The correct detergent is used for the cleaning method in use
	10. All staff use suitable protective equipment
	11. Equipment is transported to the decontamination area using a rigid, durable, leak-proof container that has a tight-fitting lid and is easy to clean and disinfect
	12. Hand pieces are cleaned as specified by the manufacturers' instructions
	13. Instruments are rinsed thoroughly following cleaning
	14. Disposable, non-linting towels are used to dry instruments immediately after rinsing
	15. All instruments are inspected with an illuminated magnifier every time after you clean
	16. Written policies on cleaning instruments within the practice are followed

1. Behaviour GDP: Dentists were asked to self-report their current practice relating to five dentist-level behaviours (see Table 1) on a four-point scale ('What is your current decontamination practice? Do you....rarely/never, sometimes, usually, always'). Responses for each behaviour were dichotomized into two categories: 'always doing the behaviour' (always = 1) and 'not doing the behaviour' (rarely/never, sometimes, usually = 0), then summed to create a score out of five.

2. Behaviour Practice: Given the dentist has the final responsibility of the performance of practice-level behaviours, we used certainty as a proxy for individual performance, by asking them to report on a seven-point scale how sure they were that each of the 11 practice-level behaviours were being performed ('In your practice how sure are you that...Not at all Sure (1) Very Sure (7)). Responses for each behaviour were dichotomized into two categories: 'very sure the behaviour is performed' (very sure (7) = 1) and 'not sure' (1 to 6 = 0), then summed to create a score out of 11.

3. Behaviour Overall: Behaviour GDP and behaviour practice scores were summed to create a score out of 16.

Higher scores denote better decontamination practice, in terms of more required behaviours being performed.

#### Secondary outcome measures

These measures follow theory operationalisation protocols [16,20].

#### Behavioural intention

For each of the 16 decontamination behaviours, participants were asked to respond on a seven-point scale to the following: How motivated are you to change your current practice in relation to.... ('Not at all' to 'Very Much'). 'Intention: GDP' was the mean score of items relating to the five dentist-level decontamination behaviours. 'Intention: Practice' was the mean score of items relating to the practice-level decontamination behaviours. 'Intention: All' was the mean score of all items. Higher scores denote greater intention to perform best decontamination practice.

#### Attitude

Attitude was assessed by asking participants to respond on seven-point scales to the following: 'How important; how necessary; and how practical are each of the following procedures' ('important' to 'unimportant'; 'necessary' to 'not at all necessary'; 'practical' to 'not at all practical')**'**. 'Attitude: GDP' was the mean score of items relating to the dentist-level decontamination behaviours. 'Attitude: Practice' was the mean score of items relating to the practice-level decontamination behaviours. 'Attitude: All' was the mean score of all the attitude items. Higher scores denote more positive attitude toward performing best decontamination practice.

#### Perceived behavioural control (PBC)

For each of the 16 decontamination behaviours, participants were asked to respond on a seven-point scale to the following: How difficult is it to.... (difficult to not at all difficult). 'PBC: GDP' was the mean score of items relating to the dentist-level decontamination behaviours. 'PBC: Practice' was the mean score of items relating to the practice-level decontamination behaviours. 'PBC: All' was the mean score of all PBC items. Higher scores denote higher perceived control over performing best decontamination practice.

#### Plans to change current practice

Dentists were asked whether they had plans in place to change their current practice in relation to the 16 outcome decontamination behaviours. Responses were dichotomized into 'have plan' (score = 1) and 'no plan' (score = 0), and then summed. Higher scores denote more plans in place to change current practice.

#### Procedure

The development of the postal questionnaire was informed by 16 semi-structured, qualitative interviews (of approximately 35 minutes), which were conducted by telephone with dentists randomly identified from the Scottish Dental Practice Based Research Network. The results are presented in Table 2. No one belief was mentioned by all participants. Only three dentists raised patient safety as an issue. All of the participants commented that they thought it would be generally be too difficult to fully implement best decontamination practice as cited in the guidance document. While 70% of participants thought that they may change something in their practice as a result of reading the guidance, there was little agreement about what they would change (<4). All participants thought they needed outside help, financial and or advice, to fully implement the guidance. A content analysis grouped all responses into TPB domains (see Table 2), and the results were validated by five independent judges (consisting of dentists and researchers unfamiliar with psychological models) achieving an outstanding index of inter-rater reliability of 80% [21]. Because no participant spontaneously identified any group or person as putting pressure on them to implement the guidance, subjective norm was not assessed in the postal questionnaire.

**Table 2:** Identified barriers and facilitators of adhering to SDCEP decontamination guidance

Interview Questions to identify Barriers		
1. Are there any aspects of the SDCEP Guidance document that you think would be particularly challenging for you or your practice to implement? Why?		
		
2. What do you feel are the disadvantages of the guidance (to you/your practice/to patients)?		

Responses	N/16	Theory variable

1. Setting up a decontamination area (difficult to find space/costly)	10	PBC
2. Purchasing/storing approved cleaning equipment (expensive equipment/expensive and difficult to change practice layout)	10	PBC
3. Validation, testing and maintenance of cleaning equipment (don't know how, difficult to do)	8	PBC
4. Finding time required (difficult to find the time to follow procedures/reduces time for patient appointments)	7	PBC/Attitude
5. Difficult to follow Guidance material (needs more clarification)	7	PBC
6. Transportation of equipment from one area to another (difficult/unnecessary fuss)	6	PBC
7. Will result in staff being unhappy/Staff will be resistant	4	Attitude
8. Will be stressful to follow procedures	3	Attitude
9. Decontamination procedures are overkill	3	Attitude

Interview Questions to identify Facilitators		

3. What would help you put the SDCEP guidance into practice?		
4. What do you feel are the advantages of the guidance to you/your practice/to patients?		

Responses	N/16	Theory variable

1. Avoid legal implications (Inspectors would not shut down the practice; reduce patients reasons to sue)	7	Attitude
2. May increase patient's confidence in the practice (fulfilling standards)	6	Attitude
3. Patient safety will be enhanced	3	Attitude

N/16 = Number of dentists out of the total 16 participants who expressed this belief; theory variables are from the theory of planned behaviour [19]; PBC = perceived behavioural control.

A power calculation suggested that a minimum sample of 129 dentists was required to detect a difference in R-squared of 0.10 with significance level of 5% and 90% power for four predictor variables in a multiple regression equation [22]. Because previous surveys of this population suggested a likely response rate of approximately 60%, two-hundred questionnaires were sent to a random sample of dental practices throughout Scotland, identified from Practitioner Services Division (PSD) Management Information Dental Accounting System database. A reminder letter with a second questionnaire was sent to non-responders two weeks later. Four weeks later, a postcard reminder was sent to the remaining non-responders.

### Statistical analysis

Statistical significance was based on two-sided tests with p ≤ 0.05 as the criterion. Measures were tested for internal consistency using Cronbach's alpha. The individual and practice-level subscales were to be combined into a single measure only if Cronbach's alpha exceeded 0.60. The relationship between predictive and outcome variables were examined using Pearson correlations and multiple regression analyses.

## Results

### Response rate and participants

Out of the 200 questionnaires posted, three were returned as undeliverable. 113 dentists returned completed questionnaire, giving a response rate of 57% (113/197). The final sample profile was: 70% male, qualified on average for 18 years (SD = 9.9), worked full time (mean (SD) sessions per week = 8.4 (2.2)), with an average practice list size of 4,532 (2,987). 12% were (or had been) a vocational trainer. Number of other dentists in the practice ranged from zero (N = 13) to 10 (N = 2). On average, there were two other dentists in the practice, four dental nurses, one hygienist, and one receptionist.

The representativeness of the study participants was examined by comparing their demographics with the available demographics of the 2006/07 Management Information Dental Accounting System database, which shows 60% of dentists were male and qualified on average for 18 yrs (this was calculated from the available information of: average age = 41/average age qualified = 23). Furthermore, the demographics of this sample was compared with an independent, randomly selected sample from the Scottish Dental Practice Board Register (N = 214) who participated in a postal study examining intra-oral radiograph ordering [19]. There were no significant differences in gender (χ^2^(1,323) = 0.18, p = 0.67); number of other practitioners in their practice (t(1,317) = -0.10, p = 0.92); years qualified (t(1,319) = 0.28, p = 0.78); number of sessions worked per week (t(1,321) = -1.29, p = 0.19); or list size (t(1,266) = -0.65, p = 0.51).

### Should an implementation intervention be developed?

No dentist reported complying with all 16 decontamination behaviours. On average, dentists reported complying with 10 (SD = 3) decontamination behaviours. Only 15% (17/113) of dentists reported they were complying with all five key dentist-level behaviours. On average, dentists were complying with three (SD = 1) out of the five dentist-level behaviours. The least performed of these was working in a clutter-free environment (Table 2). At the practice level, only 2% of dentists reported that they were sure that their practice was complying with all 11 key behaviours. On average, dentists reported that they were fairly to very sure that their practice was complying with seven (SD = 2) out of the 11 practice level behaviours. They were least sure about whether instruments were inspected under an illuminated magnifier (Table 3).

**Table 3:** Results of the Postal Survey (N = 113): Self-report current practice and plans to change current practice

*In your current infection control/decontamination practice, do you*:	Responses No (%)	Do you plan to change? Yes (%)
Remove hand and wrist jewellery at the start of each session	52%	22%

Clean hands before putting on gloves	37%	14%

Change gloves before seeing **each **patient	3%	3%

Use single use items only once	16%	6%

Work in a clutter – free environment	54%	18%

*In your practice are you sure that:*		

Decontamination equipment is used in accordance with the manufacturers' instructions	19%	6%

Testing of decontamination equipment takes place at the correct intervals	27%	10%

Decontamination activities take place in a dirty to clean workflow	23%	9%

The correct detergent is used for the cleaning method in use	19%	11%

All staff use suitable protective equipment	34%	21%

Equipment is transported using a rigid, durable, leak-proof container that has a tight-fitting lid and is easy to clean and disinfect	52%	22%

Hand pieces are cleaned as specified by manufacturers' instructions	17%	10%

Instruments are rinsed thoroughly following cleaning	18%	15%

Disposable, non-linting towels are used to dry instruments immediately after rinsing	66%	26%

All instruments are inspected with an illuminated magnifier every time after you clean	93%	22%

Written policies on cleaning instruments within the practice are followed	30%	13%

Despite all 16 behaviours showing scope for compliance improvement, only one behaviour (changing gloves before seeing each patient) showed a match between the percentage of dentists who should be changing (percentage currently not performing best practice) and the percentage of dentists who planned to change their current practice (Table 3).

### Can the theory of planned behaviour (TPB) be applied to decontamination practice?

Variables from the TPB were significantly correlated with dentist-level, practice-level and overall decontamination practice (Table 4). Intention was not correlated with decontamination behaviours and none of the attitude or perceived behavioural control measures were significantly correlated with an intention measure. Further investigation revealed that the measure of intention had a severely bimodal distribution at the extremes (scores ≤2 or ≥6), with 57% of dentists reporting that they were very motivated to change their current decontamination practice in line with the guidance (scoring ≥4).

**Table 4:** Results of the Postal Survey: Descriptive statistics and Pearson Correlations showing beliefs predicting self-report current decontamination practice

Measure	Descriptive statistics			Pearson Correlation Coefficients		
	Alpha	Range	Mean (SD)	Behaviour: GDP	Behaviour: Practice	Behaviour: Total

Attitude: GDP	0.84	3–7	6.2 (0.8)	0.68***	0.41***	0.54***

Attitude: Practice	0.92	4–7	5.9 (0.7)	0.52***	0.57***	0.59***

Attitude: All	0.93	3–7	5.9 (0.7)	0.61***	0.55***	0.62***

PBC: GDP	0.67	1–7	6.0 (1.0)	0.49***	0.33***	0.43***

PBC: Practice	0.87	2–7	5.3 (1.2)	0.42***	0.49***	0.53***

PBC: All	0.88	2–7	5.5 (1.0)	0.46***	0.50***	0.56***

Intention: GDP	0.92	1–7	3.7 (2.3)	0.03	0.03	0.06

Intention: Practice	0.97	1–7	3.7 (2.1)	0.07	0.13	-0.13

Intention: All	0.97	1–7	3.7 (2.1)	0.05	0.09	-0.12

Possible score for all measures = 1 to 7; Alpha = Cronbach's alpha; Behaviour: GDP = Self reported current practice relating to five dentist-level decontamination behaviours from SDCEP guidance document; Behaviour: Practice = Self reported current practice relating to 11 practice-level decontamination behaviours from SDCEP guidance document; Behaviour: Total = Self reported current practice relating to all 16 decontamination behaviours (See Table 1);*p < 0.05;** p < 0.01; ***p < 0.001; The Cronbach's alpha for the outcome measures were: Behaviour:GDP = 0.36; Behaviour: Practice = 0.78; Behaviour: Total = 0.79

When all variables that were significantly correlated with decontamination practice were entered into a stepwise regression analysis, attitude explained 36% of the variance in self-reported decontamination practice (Model 1, Table 5). The regression analysis was repeated for the individual attitude items. Two attitude items explained 30% of the variance in decontamination practice (Model 2, Table 5). The more necessary the dentists believed behaviours to be, the more behaviours they themselves performed. Also, how sure dentists were that decontamination behaviours were being performed in the practice was related to how practical they judged the behaviours to be.

**Table 5:** Results of the explorative stepwise regression analyses identifying beliefs accounting for variance in performing decontamination behaviour

**Model 1: All Predictive**						
**Predictive Variables**	**Entered**	**B**	**Beta**	**Adj. R**^**2**^	**df**	**F**

Attitude: GDP, Attitude: Practice, PBC: GDP, PBC: Practice	Attitude: Practice Attitude: GDP	1.75 1.10	0.41*** 0.26**			
				0.36	2,105	30.92***

**Model 2: All elements of Attitude**						

**Predictive Variables**	**Entered**	**B**	**Beta**	**Adj. R**^**2**^	**df**	**F**

Important: GDP; Necessary: GDP, Practical: GDP, Important: Practice, Necessary: Practice, Practical: Practice	Necessary: GDP Practical: Practice	1.56 0.80	0.38*** 0.28**			
				0.30	2,106	24.24***

B = Unstandardized coefficient; Beta = Standardized coefficient;* p < 0.05;** p < 0.01; ***p < 0.001

Dependent Variable: Self reported current decontamination practice relating to all 16 behaviours (Behaviour: Total) identified from the Behaviour Elicitation Study

## Discussion

The results of the postal survey suggest that there is indeed scope for enhancing the implementation of the SDCEP guidance with a further intervention. Not a single participant reported complying with the document in total. The discrepancy between self-report current practice and best decontamination practice, coupled with a compensating lack of plans to change (Table 3), further support the need for an intervention to encourage the implementation of the decontamination guidance in Scotland.

The postal survey also provided support for the applicability of the TPB to decontamination behaviours. All but one of the theory components acted in line with theoretical predictions. Dentists who had a more positive attitude toward decontamination best practice reported performing significantly more decontamination behaviours. Dentists who perceived that they had more control over performing best practice, in terms of being able to overcome barriers, reported performing significantly more decontamination behaviours. These relationships held whether the outcomes and predictors were at the dentist level or the practice level. Although a significant correlation is not evidence of a causal relationship, it is a necessary precursor of one. In particular, the results suggest that increasing dentists' beliefs in the necessary and practical nature of decontamination behaviours may encourage their implementation of the guidance. Applying this theoretical model to decontamination behaviours allowed the identification of these variables as possible mediators of decontamination best practice, providing likely targets for an implementation intervention.

In contradiction to the theoretical expectation, the measure of intention was neither significantly correlated with self-reported performance of decontamination behaviours, nor was it associated with other variables in the theory. Despite its theory-driven operationalisation, it is possible that this was an artefact of asking about multiple behaviours, because the TPB is usually applied to predicting a single behaviour. Although this did not appear to be a problem for the other theory components, our intention measure may have been highly sensitive to this issue, particularly if dentists viewed some of the decontamination behaviours as not under their volitional control (the TPB model explains behaviours within the control of the individual). This perception was apparent in the pilot study, where all participants stated that they needed outside help to fully implement the guidance. However, none of the recommended decontamination behaviours on the best practice list are, in reality, non-volitional. The erroneous perception that any of them are can be viewed as a barrier that could be addressed when targeting dentists' attitudes and perceptions of control. This suggests that the TPB can still be considered an appropriate model on which to base an intervention to influence decontamination best practice.

Nevertheless, a TPB- based intervention would focus on influencing pre-motivational elements related to behaviour in generally unmotivated people. The bimodal distribution of intention at the extremes demonstrated that our sample of participants were almost equally split between dentists who were completely unmotivated to implement best decontamination practice or else highly motivated. This result suggests that targeting TPB components would only be the best strategy for half of our sample. If this represents a true split in the larger population, then a different strategy is needed for dentists who were already very motivated to change their current decontamination practice in line with the guidance. For this proportion of the population, it would be more appropriate to design an intervention using a model that focuses on post-motivational elements, translating 'good' intentions into action.

Implementation intention theory is just such a theory. In this model, the main component influencing behaviour is action planning. This theory proposes that the likelihood of performing a behaviour can be increased by making an explicit action plan about when and where you intend to perform it [22-26]. Action plans are not proposed to work by increasing motivation, as are attitude and perceived behavioural control in the TPB. They are proposed to work by setting up environmental cues to remind an individual to perform the behaviour. Repeatedly being performed in response to the cue increases the likelihood that a behaviour may become a 'good' habit. Like the TPB, implementation intention theory has been used to successfully influence the behaviour of individuals and has been specifically associated with other evidence-based dental behaviour in previous studies [19,27]. Some support for including implementation theory in the design of an implementation intervention is provided by the notable lack of plans in place to change decontamination behaviours (Table 3). This suggests that asking already motivated dentists to formulate action plans may encourage a change in their current practice.

In summary, it does appear that an implementation strategy is required to encourage the implementation of the decontamination guidance. It also appears that the strategy will need to account for both pre- and post-motivational elements. There was some support for using the TPB to design a strategy to encourage motivation to implement the guidance in a proportion of the population sampled. The results of the postal study also suggested that a complementary strategy may need to be incorporated into an intervention – one that uses action planning to encourage the implementation of the guidance by dentists who were already motivated to do so, yet were not translating their intention into their practice.

The results of the preliminary interviews suggested that it would be difficult to unravel what would specifically help even a small number of dentists overcome the barriers they raised to implementing the decontamination guidance. The postal study confirmed that there was also variation in what the larger sample of dentists believed they should change, what they felt able to change, and what they were willing to change. These results provide some explanation of previous and current poor decontamination practice. They also suggest that an intervention that has the greatest chance of influencing the implementation of decontamination behaviours will need to have a format elastic enough to consider the very disparate concerns, motivation, and behaviour of each dentist and practice.

One way for this to be achieved is to design the intervention in the form of a 'tailored' support visit, where a researcher could assist the practice teams to identify behaviours from the decontamination list that they need to better implement. They could then use established methods to target theoretical variables. For example, techniques to enhance perceived behavioural control (changing can't to can) are identifying and changing the external barriers and facilitators of behaviour, as well as increasing the individual's skills to overcome perceived barriers. Techniques to encourage a more positive attitude (changing won't to want to) include providing information about behavioural consequences (e.g. risk), verbal persuasion, and positive feedback in relation to specific decontamination behaviours. Techniques to help individuals formulate action plans (addressing the intention-behaviour gap) include setting goals, creating an explicit undertaking about who, where, and when a specific decontamination behaviour will be performed, or missing equipment will be purchased, as well as progress monitoring and the provision of social support.

The cross-sectional nature of this research precludes conclusions about cause and effect; therefore caution is warranted in making generalizations about how effective this intervention will be on actual practice. Also, it is possible that there may be a selection bias, with study participants only representative of dentists in Scotland – or even of dentists who participate in studies in Scotland – that may also influence the effectiveness of this intervention if more generally applied. Nevertheless, a major strength of this study is the qualitative preparatory research that went into the design of the questionnaire. In helping to create an outcome measure, stakeholders were impelled to identify what the guidelines were asking all dentists in Scotland to do – not just the dentists in our sample. Having greater clarity about what is required provides a means of assessment that is applicable beyond our study. The focus on psychological theory ignores possibly valuable other approaches, such as organisational, political, and economic incentives. Nevertheless, it also provides depth and focus that may be generalisable across different behaviours as well as different populations, and takes advantage of decades of research specifically into the antecedents and methods of behaviour change.

## Conclusion

Considerable resources are currently devoted to encouraging clinicians to implement evidence-based practice using interventions with erratic success records, or no known applicability to a specific clinical behaviour, selected mainly by means of researchers' intuition or optimism. Conducting a developmental survey enabled the identification of an intervention format, mechanism, and targets with the greatest likelihood of success of increasing the implementation of decontamination guidance. The methodology used to develop this implementation intervention is not limited to the decontamination issue or to a single segment of primary care. This approach is in accordance with the preliminary stages of the framework for evaluating complex interventions suggested by the medical research council [28]. The next phases of this work are to test the intervention feasibility and evaluate its effectiveness in a randomised control trial.

## Competing interests

The authors declare that they have no competing interests.

## Authors' contributions

DB contributed to the scientific development, analysis and interpretation of the study; authored drafts and approved the final version of the paper; LY and HC contributed to the scientific development, administration, analysis, interpretation of the study, and approved the final version of the paper; IB, CR, and JC contributed to the scientific development, conduct, analysis, interpretation of the study, and approved the final version of the paper.
